# An Analytic Study on the Effect of Alginate on the Velocity Profiles of Blood in Rectangular Microchannels Using Microparticle Image Velocimetry

**DOI:** 10.1371/journal.pone.0072909

**Published:** 2013-08-30

**Authors:** Katie L. Pitts, Marianne Fenech

**Affiliations:** 1 Chemical and Biological Engineering, University of Ottawa, Ottawa, Ontario, Canada; 2 Mechanical Engineering, University of Ottawa, Ottawa, Ontario, Canada; University of Arizona, United States of America

## Abstract

It is desired to understand the effect of alginic acid sodium salt from brown algae (alginate) as a viscosity modifier on the behavior of blood *in vitro* using a micro-particle image velocimetry (µPIV) system. The effect of alginate on the shape of the velocity profile, the flow rate and the maximum velocity achieved in rectangular microchannels channels are measured. The channels were constructed of polydimethylsiloxane (PDMS), a biocompatible silicone. Porcine blood cells suspended in saline was used as the working fluid at twenty percent hematocrit (H = 20). While alginate was only found to have minimal effect on the maximum velocity and the flow rate achieved, it was found to significantly affect the shear rate at the wall by between eight to a hundred percent.

## Introduction

Viscosity modification of blood flows is of importance to biomedical research for many reasons. In a clinical setting, modifying the viscosity of blood can be desired in order to reverse non-standard physiology, or to incorporate a medical benefit such as drug delivery [1]. While blood may be considered a homogeneous fluid for some macroscale studies, at the micro scale blood must be considered a suspension of flexible formed elements such as red blood cells (RBCs), white blood cells (WBCs) in a Newtonian matrix of plasma. Blood viscosity modification is frequently done by introducing other flexible elements in the form of polymers such as dextran, polyethylene glycol (PEG), polyethylene oxide (PEO), warfarin, heparin, and pluronics [1]. Often the goal of these polymers is to thin the blood, avoid thrombosis or to decrease aggregation. Warfarin and heparin are common blood thinners used in modern medicine. Pluronics have been shown to decrease low shear blood viscosity and have been used in clinical trials as a viscosity modifier for sickle cell anemia [2]. PEO has been shown to reduce the cell-free layer, the naturally occurring red blood cell depleted zone at the wall of a microchannel or vessel, in some concentrations [3]. Dextran, PEO and PEG can induce the aggregation of RBC if the correct molecular weight or concentration. In general, long-chain polymers induce aggregation and short-chain polymers inhibit aggregation or have no effect [3]. Alginic acid, alginate, is a straight-chain polyuronic acid with hydrophilic properties. Alginate is a polymeric viscosity modifier that has many uses in industry including biomedical applications such as tissue engineering and drug encapsulation [4]. Alginate, a derivative of brown algae cells, is biocompatible and biomimetic [5]. Additionally alginate has been suggested to have applications within *in vivo* or *in vitro* blood research [6].

There have been conflicting reports on the behavior of alginate when combined with blood. The addition of sodium alginate has been found to greatly increases the rate of sedimentation of human and horse red blood cells (RBCs) and can be lethal for cats, rabbits and mice when injected *in vivo* [7]. Other research found that long-chain polymers of alginate could cause renal failure, while short-chain polymers of alginate are thought to not cause these effects [4]. Alginate has also been found to affect the aggregation of RBCs in a dose-dependent manner [4]. Significant research has gone into using alginate and other plasma expanders to change the viscosity of the blood during extreme hemodilution, to increase perfusion, maintenance of blood pressure, and in the avoidance of hemorrhagic shock [8]–[13]. Research has also suggested *in vitro* application of alginate to decrease large cell settling [6]. Alginate cannot both decrease settling and increase aggregation, but it is possible that settling effects are dose or chain length dependent as well, which could explain differences in results. The benefits of alginate as a plasma expander and viscosity modifier have been demonstrated but the flow profiles of blood modified by alginate are necessary to fully describe the phenomenon present. To that end, the actual effect of the alginate on the velocity profile must be measured in a repeatable manner.

Measuring velocity profiles in blood flows at the range of the microcirculation is a nontrivial research problem in and of itself. For any scale flow, a velocity profile cannot be measured directly, but must be reconstructed from flow data. These profiles are generally presented as an average velocity profile. There are several approaches one may take in the medical imaging of blood flows. Magnetic resonance (MR) imaging, photoacoustic (ultrasound) Doppler, optical coherence tomography, and particle image velocimetry (PIV) are all currently used methods [14]–[17]. At the scale of the microcirculation, not all imaging techniques can be used as there are many further challenges associated with micron level medical imaging. When using optical techniques in larger flows, single planes may be illuminated; in micron scale flows the entire volume must be illuminated. Micro-particle image velocimetry (µPIV) was chosen due to its ability to measure fields of velocity which can be averaged for a velocity profile, over single particle flows like particle-tracking methods, and its accuracy at the micron scale despite requiring volume illumination.

The purpose of this paper is to demonstrate the effect of alginate on *in vitro* microblood flows, as demonstrated by the resulting flow rate, maximum centerline velocity, and shear rate developed at the wall of the channel. Additionally, it is desired to quantitatively compare change in the shape of the velocity profile at the micro scale and to compare findings with conflicting reports in the literature. It was hypothesized that the sodium alginate will affect the velocity profiles measured. Flow rate and alginate addition were studied at 20% hematocrit in polydimethylsiloxane (PDMS) microchannels. PDMS channels have previously been shown to be effective in the study of blood [18]–[19]. PDMS is a biocompatible polymer and currently is one of the primary reference materials for the evaluation of other biomaterials [20].

## Materials and Methods

### Channel Design and Fabrication

Rectangular (140 µm wide by 40 µm tall) PDMS channels were fabricated of Slygard-184 (Dow-Corning, USA) in a cleanroom following established soft lithography techniques [21]–[23]. PDMS is an excellent choice to mimic the behavior of the vascular network, which includes its deformability. The tubing is necessarily flexible in order to integrate the microchip into the µPIV system. Both of these factors lead to a compliance in the system. The PDMS channels were bonded to unused, standard glass microscope slides using oxygen plasma. Prior to plasma bonding, slides were soaked in acetone, rinsed in isopropanol then rinsed in methanol to ensure they were free from contamination. Slides were dried using compressed nitrogen. PDMS channels were cleaned with scotch tape to ensure they were free from dust. As fully developed flow was desired, the entrance length was calculated using an experimentally verified technique for microfluidic channels [24]. Entrance lengths for the channel were found to be on the order of the hydrodynamic radii of the channels. The data were taken at least 100 times the entrance lengths distance from the inlet of flow to ensure avoidance of entrance effects.

### Sample Preparation

A healthy porcine blood sample was obtained from an accredited facility. Porcine blood was chosen since RBCs in porcine blood aggregate in the same manner as human blood and the shear force required to break up aggregates is the closest to humans [25]. The whole blood sample was treated with an addition of 1 g of ethylenediaminetetraacetic acid (EDTA) per 1 L of blood as an anticoagulant. In order to obtain the RBCs the whole blood was centrifuged 3 times for 10 minutes each at 3000 RPM to separate the RBCs from the plasma and any other particles. After each centrifugation the plasma and remaining particles were removed. Small amounts of phosphate buffered saline (PBS) were added after the first and second centrifugation to the remaining RBC. After the third centrifugation the RBCs were suspended in either PBS or alginate solutions in PBS obtained by seeding low viscosity alginic acid sodium salt powder (Sigma-Aldrich, Canada) into PBS in concentrations of 4 mg of alginate per 1 mL [6]. Alginate solutions were fully dissolved in PBS before addition of RBCs. Fluorescent tracer particles (Microgenics,USA) were seeded into the resulting blood fluids at 27 µL of particles per 1 mL of solution.

The viscosity of the alginate in saline and in saline with RBC were tested in shear using a microviscometer (RheoSense, USA). A range of shear rates from 100 1/s to 1000 1/s were used for all samples. The 4 mg of alginate per 1 mL of PBS yielded viscosity measurements of 24.45 mPa-s to 17.29 mPa-s. This is a slight shear-thinning effect. The addition of RBCs increased the viscosity to 38.34 mPa-s to 19.83 mPa-s. A viscosity of 3.3 cP (3.3 mPa-s) is often reported for whole blood, while for plasma alone a viscosity of 1.1 to 1.6 cP [26].

### Ethics Statement

The porcine blood samples used in this research were collected with the authorization and approval of the Ontario Ministry of Agriculture, Food and Rural Affairs, Food Inspection Branch.

### Design of Experiment

In order to test the effect of the presence of alginate at various flow rates, RBC suspended in PBS at 20% hematocrit (H = 20) were tested at three flow rates, Q = 10, 25, 40 µL/hr. The tests were repeated at the same flow rates for H = 20 with the alginate-seeded PBS. For all data sets triplicates of each data point were taken. The average value of the three measurements for the maximum velocity, flow rate, and the maximum shear rate at the wall are reported in Table 1.

**Table 1:** Results of the various test conditions for flow rate, shear rate and maximum centerline velocity where H is the hematocrit, 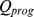 is the programmed flow rate of the syringe pump, 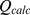 is the average flow rate calculated from the measured velocity profile, SD is the standard deviation, 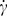 is the average measured shear rate at the wall, and 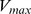 is the average maximum velocity.

Test	H	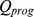	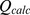	SD	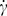	SD	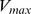	SD
(fluid)	(%)	(*µ*l/hr)	(*µ*l/hr)		(1/s)	(mm/s)	(mm/s)	
blood	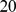	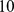	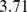	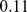	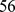	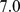	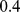	0.000012
with alginate	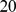	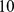	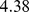	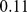	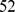	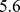	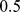	0.000043
blood	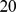	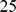	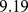	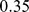	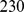	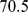	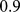	0.000012
with alginate	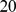	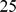	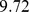	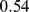	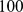	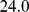	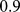	0.000018
blood	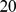	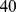	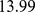	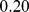	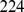	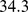	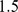	0.000061
with alginate	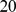	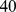	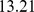	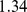	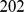	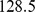	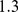	0.000101

Microchannels are PDMS with dimensions of 40 *µ*m×140 *µ*m.

### Velocity Profile Measurement

Accurate measurements of the velocity profile and maximum centerline velocity of blood flow in microfluidic channels can be made using a µPIV system. In µPIV, a microscale flow is seeded with tracer particles, in this case fluorescence labeled polystyrene particles 1 µm diameter, and two partially overlapping images of the flow are taken with a fast frame rate. Following masking and possible image pre-processing, cross-correlation is applied between to two images to obtain velocity vectors. These velocity vectors are averaged in time and space to obtain an indicative velocity profile in a microchannel. The central plane is found by measuring the velocity profile across the height of the channel and finding the maximum. Due to the large relative size of RBC to the channel dimensions in microflow, there is a large depth of correlation (DOC) in using the RBC themselves as the tracer particles for the cross-correlation, the mathematical method in which the velocity vectors of the flow are calculated. This DOC can be as large as the channel itself [27]. The out of focus particles have been demonstrated to be the greatest problem with the DOC [28]. To minimize the DOC problem, seeding with micron-scale fluorescing tracer particles have been described [17], [29] and this approach was used here. Data processing was done following the image overlapping technique of Nguyen *et al.* as applied to blood by Pitts *et al.* [30]–[31].

**Figure 1:**
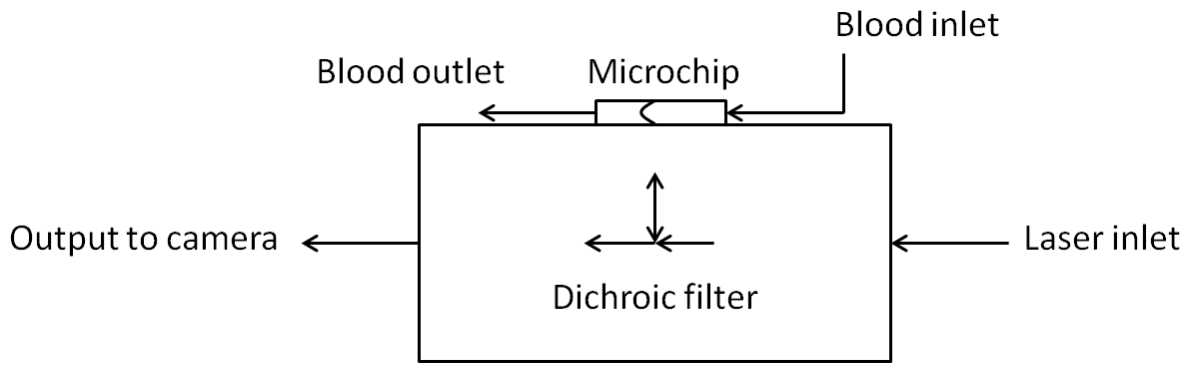
Diagram of experimental µPIV set-up showing the optical path and microfluidic component placement.

Data was obtained using the LaVision MITAS µPIV system which consists of an Nd:YAG laser and CCD camera controlled by a programmable triggering unit, a fluorescent microscope coupled with a stage moving in three axes, and a computer. A pulsed camera (Imager Intense, LaVision GmbH, Germany) was used for the cross-correlation and vector. The pulsed camera is coupled with the laser to excite the particles. A diagram of the system is shown in Figure 1. Blood was introduced to the microchips via high precision micro-syringe pump (Nexus3000, Chemyx Inc., USA).

### Calculations

The velocity profiles were obtained using cross-correlation of the µPIV data to obtain velocity vectors, which were averaged across the channel to give a single velocity profile as described in Pitts, *et al.* [31]. Shear rates given are the maximum shear rate calculated at the wall. Flow rate was calculated with a projected three-dimensional flow rate using a ratio of the experimental velocity profile to an existing theoretical, Newtonian velocity profile. The theoretical profile was calculated using Poiseuille's Law for rectangular microchannels [32]. The input flow rate of the syringe pump is used to back-calculate the pressure contribution. The width to height ratio is not great enough to constitute slit flow.
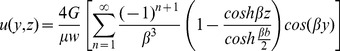
(1)where G is the pressure drop, 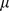 is the viscosity, 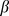 is the 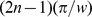, 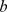 (z-direction) is the height, and 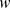 (y-direction) is the width [32]. The ratio of the experimental profile to the theoretical Newtonian flow profile was then extended into three dimensions and calculated a projected, experimental flow rate, where:



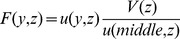
(2)In order to quantify the effect on the velocity profile, a velocity profile factor is fitted to the middle profile of each data. In flows of blood through microvessels the velocity profile can be fitted with a power law model.
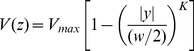
(3)where 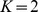 giving parabolic profile (Newtonian), 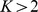 giving a blunted profile. In microhemodynamics this description is sometimes not specific enough. Koutsiaris gives a two-factor model where
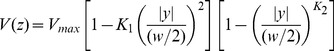
(4)with 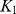 giving the bluntness near the axis and 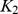 giving the bluntness near the wall [33]. This equation is fitted to all of the data. While Koutsiaris used circular channels and this work presents rectangular channels the central profile of the channel can be characterized by shape parameters regardless.

Additionally, the effect of the alginate on the shear rate at the wall is measured by calculating the shear rate for each profile.
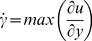
(5)


## Results and Discussion

Table 1 provides the calculated flow rate, shear rate and maximum velocity. There was almost no effect found on the maximum centerline velocity in the microchannel with the addition of alginate, at any of the programmed flow rates tested. In addition, there was little effect on the flow rate with the addition of alginate. The standard deviation of the calculated flow rates for both alginate and non-alginate samples are listed. There is a discrepancy between the calculated flow rate and the programmed flow rate of the pump. There is necessarily some compliance in the system due to the flexible nature of the PDMS and the tubing. This accounts for a portion of the discrepancy. Additionally, it is known that the PDMS deforms while flow is occurring [34]. The discrepancy in flow rate is discussed more in depth later on. It can be seen that in most cases (except the highest flow rate) the presence of alginate increases the calculated flow rate.

**Figure 2:**
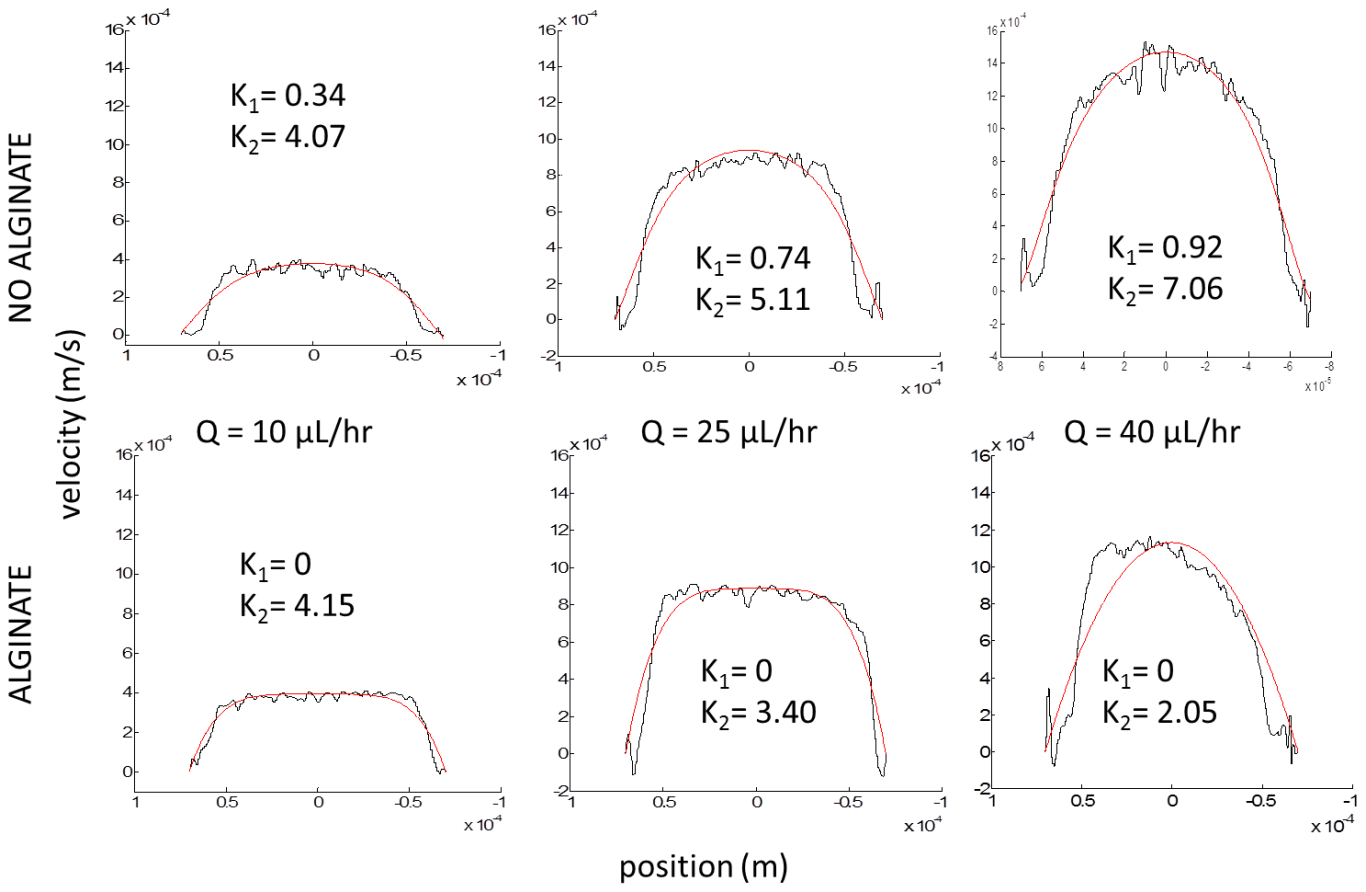
Comparison of the effect of alginate on the velocity profiles of blood with 20% hematocrit at various flow rates in a 140 µm wide, 40 µm tall PDMS channel. Black line is data and red line is fitted two-parameter K-model.

The shear rate is found to be significantly lowered by the presence of alginate at all flow rates, which can be seen visually in Figure 2, and numerically in Table 1. The standard deviations of the shear rate measurements for the alginate and non-alginate samples are listed. The shear rate can be seen to reduce by 8% at the flow rate of 10 µL/hr to an over 100% reduction at 25 µL/hr programmed flow rate. This reduction of shear rate has not been documented in literature, but has several possible implications on future research. The shear rates of the blood on the endothelial cell walls of the microcirculation regulate the uptake of nutrients and oxygen. Chiu and Chen provide an excellent overview of the current research of this nutrient uptake and shear rate behavior [35]. The addition of alginate could be used to tune the shear rate of blood solutions or blood substitutes in future research.

Table 2 gives the results of the K-fitting parameters, with the values and placed in representative profiles of Figure 2 for easier comparison between profiles. It is important to note that Figure 2 provides the middle profile of the various programmed flow rates in the syringe pump. Samples without alginate are in the top row, and samples with alginate are in the bottom row. It can be seen that for all of the cases of alginate, the 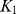 parameter goes to zero, and the 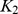 parameter decreases as the flow rate increases. 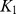 is the bluntness at the wall, and when alginate is added the flow is essentially plug-flow, and the correlation gives a valid prediction of the shape of the profile. At the case of the highest flow rate, the profile is fitted to nearly K = 2, whereas looking at the profile in Figure 2 its apparent this is not the case. As the flow rate increases, while alginate is added to the sample, the profile becomes increasingly non-uniform in all three runs and the correlation no longer gives meaningful results. This can also be seen by the severe increase in standard deviate for that sample. It more than doubles.

**Table 2:** Velocity profile characterization results of the various test conditions using two-parameter K model where H is the hematocrit, 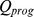 is the programmed flow rate of the syringe pump, 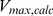 is the average calculated maximum velocity in the channel, 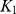 is the first shape parameter, SD is the standard deviation, and 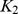 is the second shape parameter.

Test	H	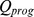	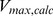	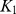	SD	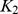	SD
(fluid)	(%)	(*µ*l/hr)	(mm/s)				
blood	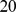	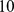	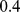	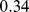	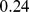	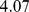	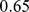
with alginate	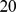	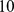	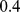	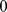	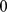	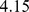	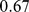
blood	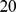	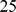	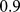	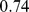	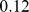	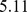	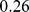
with alginate	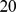	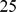	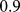	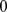	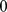	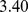	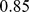
blood	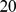	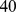	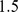	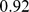	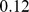	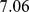	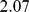
with alginate	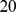	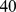	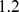	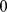	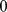	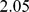	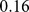

Microchannels are PDMS with dimensions of 40 *µ*m×140 *µ*m.

Single-tailed t-tests were performed on the results to ensure statistical significance. The difference in maximum velocity between the samples containing alginate and the plain samples is only significant at the highest flow rate, Q = 40 µL/hr (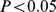). The difference between samples is not statistically significant (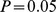), which is likely due to the small sample size. The real test on the effect of the alginate on the shape of the velocity profile is the shape parameters, 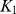 and 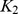. At the lowest flow rate, the difference just fails significance at 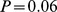, while at the higher two flow rates the difference in 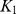 is very significant (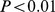). For 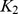 again the lowest flow rate is not a significant difference while at the higher two flow rates the difference is significant (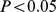). Therefore it can be concluded that the effect of the alginate is flow rate dependent.

The various viscosity modifiers used with blood such as Pluronics, PEO, PEG, and Dextran can all be used to induce or restrict aggregation. Using alginate, a naturally occurring polymer, as a viscosity modifier for blood has the same long-term goal of adjusting the bloods viscosity and aggregation. The effect of the alginate on the aggregation was not directly measured with this study, but the effect can be qualitatively evaluated. Using PBS as a medium instead of native plasma removes the aggregation ability of the blood. Microscopy images have shown how the presence of alginate affects the aggregation of the RBC [4], however all of their test suspensions contained at least some native plasma. In order to isolate the effect of the alginate from the effect of the presence of the proteins in the native plasma, here only saline was used. Research demonstrates that the RBCs deformability is not affected by the alginate and that alginate might be a useful aggregation-inducing additive to the blood [4]. Here it is demonstrated that the alginate also significantly reduces the shear rate at the wall of the channel, and alginate might also be a useful additive in that regard, and this requires further research directly comparing blood samples with and without alginate that are based on saline versus native plasma. The addition of alginate may indeed be causing aggregation, as aggregation can cause changes to the velocity profile. A more sure way to test aggregation is to use an aggregometer. A future study is planned to quantify the effect of the alginate on the aggregation of the RBCs in both saline and native plasma to support these qualitative results.

It is interesting to note that the flow rate calculated in the channel in Table 1 is less that the flow rate programmed into the pump, also listed in Table 1. It is known that bottlenecking occurs in rigid microfluidic channels and that systems also display a residence time [32]. Tabeling [32] gives a relation were the residence time of a piston-driven rigid system (much like a microsyringe) will have a residence time that varies as 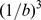, 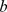 being the height of the channel. If we assume a viscosity of blood to be 3.3 cP and our system to be rigid, than the time constant for our system is on the order of many hours. However, RBCs settle out of saline solutions in a matter of minutes, thus the syringe is reloaded every 2 minutes to avoid RBC settling and steady state is never reached, thus the programmed flow rate is never achieved. We acknowledge that PDMS is not entirely rigid, and has been known to deform [34]. This deformation is minor relative to the residence time issue.

There have been reported changes in the width of the cell-free layer with the addition of polymer additives to blood [1]. In blood flows at this scale, the cell-free layer should be apparent, as well as the Fåhræus and Fåhræus-Lindqvist effects [36]. At the level of magnification used for this study, it is difficult to make an accurate measurement of the cell-free layer. Current work on this project is to increase magnification and work with the high-speed photography capabilities of the system in order to draw clearer conclusions on the effect of the alginate on the cell-free layer. Qualitatively, settling is not eliminated in the tubing and therefore the channel but was unable to be measured using this experimental configuration.

## Conclusions

Alginate has been shown to have minimal effect on the maximum velocity achieved in microflows of blood, as measured by µPIV. A significant reduction in the shear rate at the wall of the channel was found with the addition of alginate to the PBS. This could be due to the alginate-induced aggregation of the RBC as studied by Zhao *et al.* [4]. The addition of alginate could be used in future research to adjust the flow rate and shear properties of blood solutions or blood substitutes. Alginate gives a blunted velocity profile at all flow rates, so much so the edge K value goes to zero when describing the velocity profile with a two-parameter shape equation. In conclusion, it has been demonstrated that alginate can be used as a viscosity modifier in blood flows of saline and RBCs to decrease the shear rate at the wall and flatten the overall velocity profile. This behavior could be used in applications of alginate as a viscosity modifier for blood in certain medical situations.
